# Progress in site-specific cancer mortality in Canada over the last 70 years

**DOI:** 10.1038/s41598-024-56150-x

**Published:** 2024-03-07

**Authors:** Matthew T. Warkentin, Yibing Ruan, Larry F. Ellison, Jean-Michel Billette, Alain Demers, Fei-Fei Liu, Darren R. Brenner

**Affiliations:** 1 Department of Oncology, Cumming School of Medicine, University of Calgary, 3330 Hospital Drive NW, Calgary, AB Canada; 2 Centre for Population Health Data, Statistics Canada, Ottawa, ON Canada; 3 Adult Chronic Diseases and Conditions Division, Public Health Agency of Canada, Ottawa, ON Canada; 4 Institute of Cancer Research, Canadian Institutes of Health Research, Ottawa, ON Canada

**Keywords:** Cancer, Health care, Medical research, Oncology, Risk factors

## Abstract

In Canada, the absolute number of cancer deaths has been steadily increasing, however, age-standardized cancer mortality rates peaked decades ago for most cancers. The objective of this study was to estimate the reduction in deaths for each cancer type under the scenario where peak mortality rates had remained stable in Canada. Data for this study were obtained the Global Cancer Observatory and Statistics Canada. We estimated age-standardized mortality rates (ASMR, per 100,000) from 1950 to 2022, standardized to the 2011 Canadian standard population. We identified peak mortality rates and applied the age-specific mortality rates from the peak year to the age-specific Canadian population estimates for subsequent years (up to 2022) to estimate the number of expected deaths. Avoided cancer deaths were the difference between the observed and expected number of cancer deaths. There have been major reductions in deaths among cancers related to tobacco consumption and other modifiable lifestyle habits (417,561 stomach; 218,244 colorectal; 186,553 lung; 66,281 cervix; 32,732 head and neck; 27,713 bladder; 22,464 leukemia; 20,428 pancreas; 8863 kidney; 3876 esophagus; 290 liver). There have been 201,979 deaths avoided for female-specific cancers (breast, cervix, ovary, uterus). Overall, there has been a 34% reduction in mortality for lung cancer among males and a 9% reduction among females. There has been a significant reduction in cancer mortality in Canada since site-specific cancer mortality rates peaked decades ago for many cancers. This shows the exceptional progress made in cancer control in Canada due to substantial improvements in prevention, screening, and treatment. This study highlights priority areas where more attention and investment are needed to achieve progress.

## Introduction

Cancer is the leading cause of mortality globally and in Canada^1,2^. The number of cancer deaths has been steadily increasing over time, and it is estimated there was 85,100 cancer deaths in Canada in 2022, with 20,700 from lung and bronchus, 9400 from colorectal, 5500 from female breast, and 4600 from prostate cancer^2^. The steady increase in the number of cancer deaths over time can be attributed, in part, to a growing population and a shift in the age distribution of the population to older ages within which cancer is considerably more common. Despite the significant impact of cancer mortality on the Canadian population and healthcare systems, the mortality rates for most cancers have decreased considerably since the late 1980’s. Overall age-standardized cancer mortality rates for males and females peaked more than 30 years ago, and for many cancers the site-specific mortality rates have been in decline for years. The declines reflect the substantial progress made in cancer control over this time period.

There have been significant advances in our understanding of cancer etiology and biology that have enabled improvements in cancer prevention, screening and early detection, treatment strategies, molecular targeted therapies, and immuno-oncologics that have reduced cancer mortality and prevented cancer deaths. The objective of this study was to estimate the number of site-specific cancer deaths avoided in Canada in the period since age-standardized mortality rates peaked for males and females. Specifically, we aim to quantify the reduction in cancer mortality and estimate the number of potential lives saved because of the meaningful progress made in cancer control in Canada over the last several decades.

## Methods

### Sources of data

The data for this study came from two publicly-available sources, the Global Cancer Observatory (GLOBOCAN)^3^ and Statistics Canada^4,5^. Data were downloaded from GLOBOCAN using their web-based graphical user interface. Data from Statistics Canada were obtained through requests to the Web Data Services (WDS) Application Programming Interface (API).

Age- and sex-specific population counts, mortality rates, and number of observed cancer deaths were obtained from GLOBOCAN (1950–1999) and Statistics Canada (2000–2022) for each cancer site  (see Supplemental Fig. 1). Data availability by cancer site and sex are presented in Supplemental Fig. 2. See Supplemental Table 1 for site-specific cancer definitions and the corresponding International Classification of Disease, 10th revision (ICD-10) codes. Not all cancer sites were available for all years.

**Figure 1:**
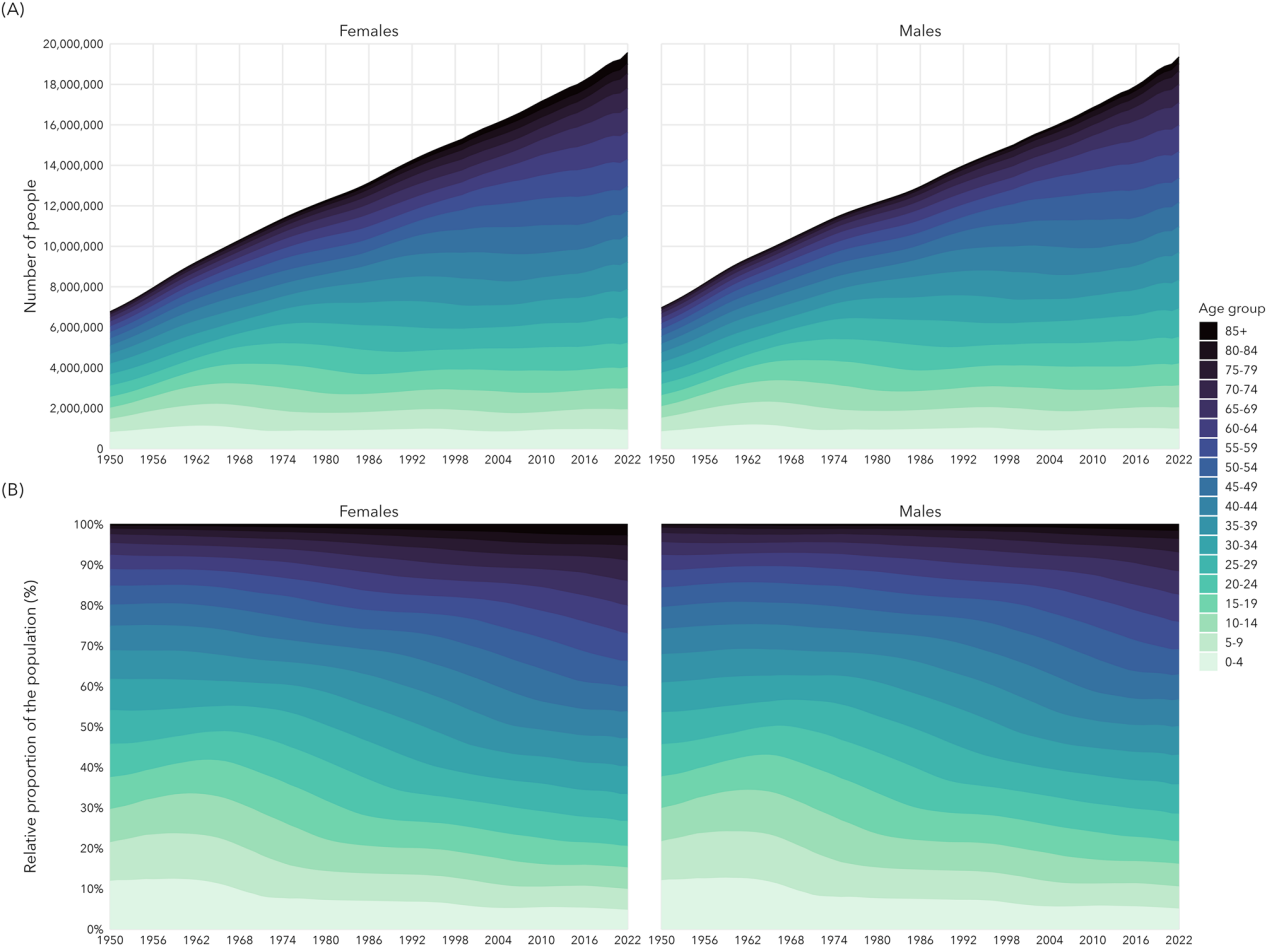
(**A**) The total number of people in Canada by sex and age group from 1950 to 2022. (**B**) The relative proportion of people in Canada in each age group and by sex from 1950 to 2022.

**Figure 2:**
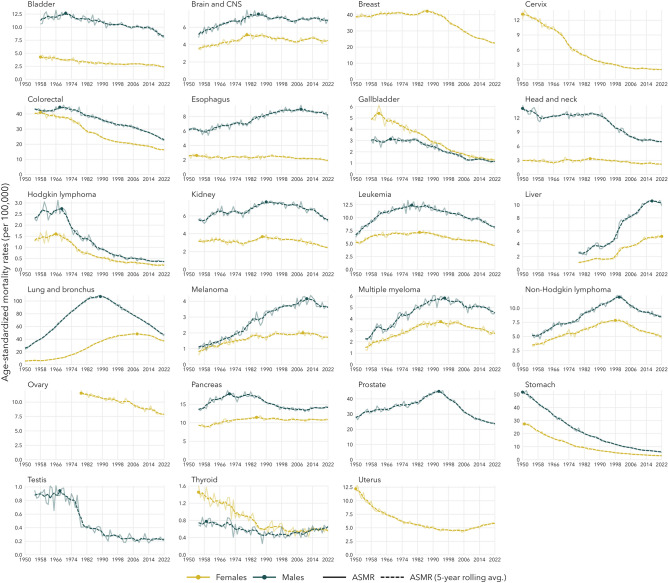
Age-standardized mortality rates from 1950 to 2022, by sex and cancer type, standardized to the 2011 Canadian standard population. Points indicate the year of peak mortality.

**Table 1:** Number of observed, expected, and avoided cancer deaths and standardized mortality ratios (SMR) for the period after age-standardized mortality rates peaked in Canada for each cancer type and sex.

	Sex	Peak Year	Peak ASMR^a^	Period	Cancer deaths			SMR^c^
					Observed	Expected^b^	Avoided	
Stomach	Males	1950	51.9	1951–2022	104,073	350,609	246,615	0.30 (0.30–0.30)
	Females	1951	27.4	1952–2022	59,281	230,191	170,946	0.26 (0.26–0.26)
Colorectal	Males	1968	44.5	1969–2022	185,562	266,481	80,949	0.70 (0.69–0.70)
	Females	1958	41.0	1959–2022	189,094	326,260	137,295	0.58 (0.58–0.58)
Lung and bronchus	Males	1989	107.0	1990–2022	336,597	509,937	173,340	0.66 (0.66–0.66)
	Females	2008	48.3	2009–2022	129,167	142,380	13,213	0.91 (0.90–0.91)
Cervix	Females	1950	13.2	1951–2022	35,185	101,311	66,281	0.35 (0.34–0.35)
Breast	Females	1987	42.2	1988–2022	174,517	238,821	64,347	0.73 (0.73–0.73)
Prostate	Males	1993	45.0	1994–2022	113,574	173,438	59,878	0.65 (0.65–0.66)
Uterus	Females	1950	12.2	1951–2022	46,939	103,633	56,705	0.45 (0.45–0.46)
Gallbladder	Males	1968	3.1	1969–2022	10,685	18,609	7,936	0.57 (0.56–0.59)
	Females	1962	5.4	1963–2022	18,759	48,022	29,263	0.39 (0.39–0.40)
Head and neck	Males	1950	14.0	1951–2022	67,537	96,371	28,834	0.70 (0.70–0.71)
	Females	1985	3.3	1986–2022	15,832	19,702	3,898	0.80 (0.79–0.82)
Bladder	Males	1971	12.6	1972–2022	53,822	72,676	18,854	0.74 (0.73–0.75)
	Females	1958	4.3	1959–2022	24,686	33,470	8,859	0.74 (0.73–0.75)
Hodgkin lymphoma	Males	1969	2.7	1970–2022	5231	18,690	13,464	0.28 (0.27–0.29)
	Females	1966	1.6	1967–2022	3802	12,915	9,121	0.29 (0.29–0.30)
Leukemia	Males	1979	12.4	1980–2022	54,070	68,197	14,146	0.79 (0.79–0.80)
	Females	1983	7.2	1984–2022	37,434	45,727	8,318	0.82 (0.81–0.83)
Pancreas	Males	1971	17.8	1972–2022	85,673	105,031	19,441	0.82 (0.81–0.82)
	Females	1985	11.5	1986–2022	67,719	68,335	987	0.99 (0.98–1.00)
Non-Hodgkin lymphoma	Males	2000	12.0	2001–2022	33,009	42,301	9,308	0.78 (0.77–0.79)
	Females	1998	7.8	1999–2022	28,861	36,412	7,565	0.79 (0.78–0.80)
Ovary	Females	1979	11.5	1980–2022	62,725	77,371	14,646	0.81 (0.80–0.82)
Kidney	Males	1990	7.6	1991–2022	30,022	35,049	5,027	0.86 (0.85–0.87)
	Females	1988	3.7	1989–2022	18,196	22,023	3,835	0.83 (0.81–0.84)
Thyroid	Males	1959	0.8	1960–2022	3655	4276	715	0.85 (0.83–0.88)
	Females	1955	1.5	1956–2022	5780	13,195	7,415	0.44 (0.43–0.45)
Brain and CNS	Males	1986	7.5	1987–2022	36,046	38,338	2,349	0.94 (0.93–0.95)
	Females	1980	5.1	1981–2022	30,164	33,648	3,484	0.90 (0.89–0.91)
Multiple myeloma	Males	1996	5.8	1997–2022	19,482	22,273	2,804	0.87 (0.86–0.89)
	Females	1994	3.8	1995–2022	16,886	19,286	2,453	0.88 (0.86–0.89)
Testis	Males	1968	0.9	1969–2022	2568	6890	4,322	0.37 (0.36–0.39)
Esophagus	Males	2008	9.0	2009–2022	21,558	24,055	2,497	0.90 (0.88–0.91)
	Females	1954	2.6	1955–2022	18,788	20,042	1,379	0.94 (0.92–0.95)
Melanoma	Males	2011	4.2	2012–2022	7697	7706	248	1.00 (0.98–1.02)
	Females	2009	2.0	2010–2022	5085	5767	682	0.88 (0.86–0.91)
Liver	Males	2017	10.6	2018–2022	10,713	11,003	290	0.97 (0.96–0.99)
	Females	–	–	–	–	–	–	–

^a^Age-standardized mortality rates are per 100,000 people based on direct standardization to the sex-specific 2011 Canadian standard population. We used a 5-year rolling average ASMR to identify the peak year of mortality.

^b^Expected cancer deaths were estimated based on applying age-specific mortality rates from the peak year to age-specific population counts for every year in the period following the year of peak mortality.

^c^Standardized mortality ratios (SMR) were estimated as the ratio between the expected and observed number of deaths. Confidence intervals (95%) were estimated using the Vandenbroucke method ^8^.

*ASMR* age-standardized mortality rate, *CNS* central nervous system, *SMR* standardized mortality ratios.

The table is ordered from top to bottom according to the descending number of cancer deaths avoided (total by site).

### Statistical analysis

All analyses described herein were performed separately for males and females. Age groups were based on five-year bins up until age 84; all ages 85 years and beyond formed the 85+ age group. Age-specific number of cancer deaths were reported directly by Statistics Canada from 2000 to 2022. We derived the age-specific number of deaths from 1950 to 1999 using GLOBOCAN crude mortality rates and population counts. For each sex and cancer site, we estimated the age-standardized mortality rates (ASMR, per 100,000) and 95% confidence intervals^6^ from 1950 to 2022, standardized to the 2011 Canadian standard population (^7^, see Supplemental Table 2), using age-specific mortality rates and population counts. The peak years of site-specific mortality rates were defined as the maximum ASMR (based on the 5-year rolling average), separately for each sex. Note that for some cancer sites, mortality rates have been declining throughout the reference period considered for this study and the peak rate identified in this study may not reflect the true peak mortality rate, which happened before 1950.

For every year in the period following the peak mortality, the expected number of cancer deaths was estimated by applying the peak-year age-specific mortality rates to the age-specific population counts for the index year. The number of avoided cancer deaths was estimated as the difference between the expected and observed number of cancer deaths in that period. We estimated standardized mortality ratios (SMR) and 95% CI^8^ based on the ratio of the observed to expected number of cancer deaths. The relative reduction in expected deaths over time was estimated as the number of avoided deaths divided by the number of expected deaths. The ratio of the relative reductions in expected deaths (males-to-females) were estimated to assess potential sex differences in preventing cancer mortality. We did not sum avoided deaths across cancer types as cancers had different peak years of mortality and multiple cancers could have developed in the same person and summations could have led to an over-estimation. All statistical analyses were performed using R version 4.2.2 (2022-10-31).

### Ethical approval

This study used publicly-available data and research ethics approval was not required.

## Results

### Age-standardized mortality rates

The Canadian population has grown from approximately 14 million people in 1950 to 39 million in 2022, growing by 1.46% each year, on average, since 1950 (see Fig. 1A). In addition, the relative age structure of the Canadian population has changed substantially over time (see Fig. 1B). Much of this growth has been due to immigration^9^, which also modifies the risk pool of the Canadian population and may lead to shifts in incidence and mortality trends in the coming years. The wave of younger individuals in the 1950’s and 1960’s has led to an older population in more recent years. Nearly 42% of the population of Canada was under 20 years of age in 1964, down to 21% in 2022.

We estimated the age-standardized mortality rates and the five-year rolling average ASMR (per 100,000 population) for site-specific cancer mortality for all years from 1950 to 2022 (where data were available). The ASMR over time are presented in Fig. 2. Among all the cancer-sex combinations assessed in this study, only liver cancer among females had not yet peaked. The peak year of mortality and the corresponding ASMR for each cancer type are presented in Table 1.

### Avoided cancer deaths

**Figure 3:**
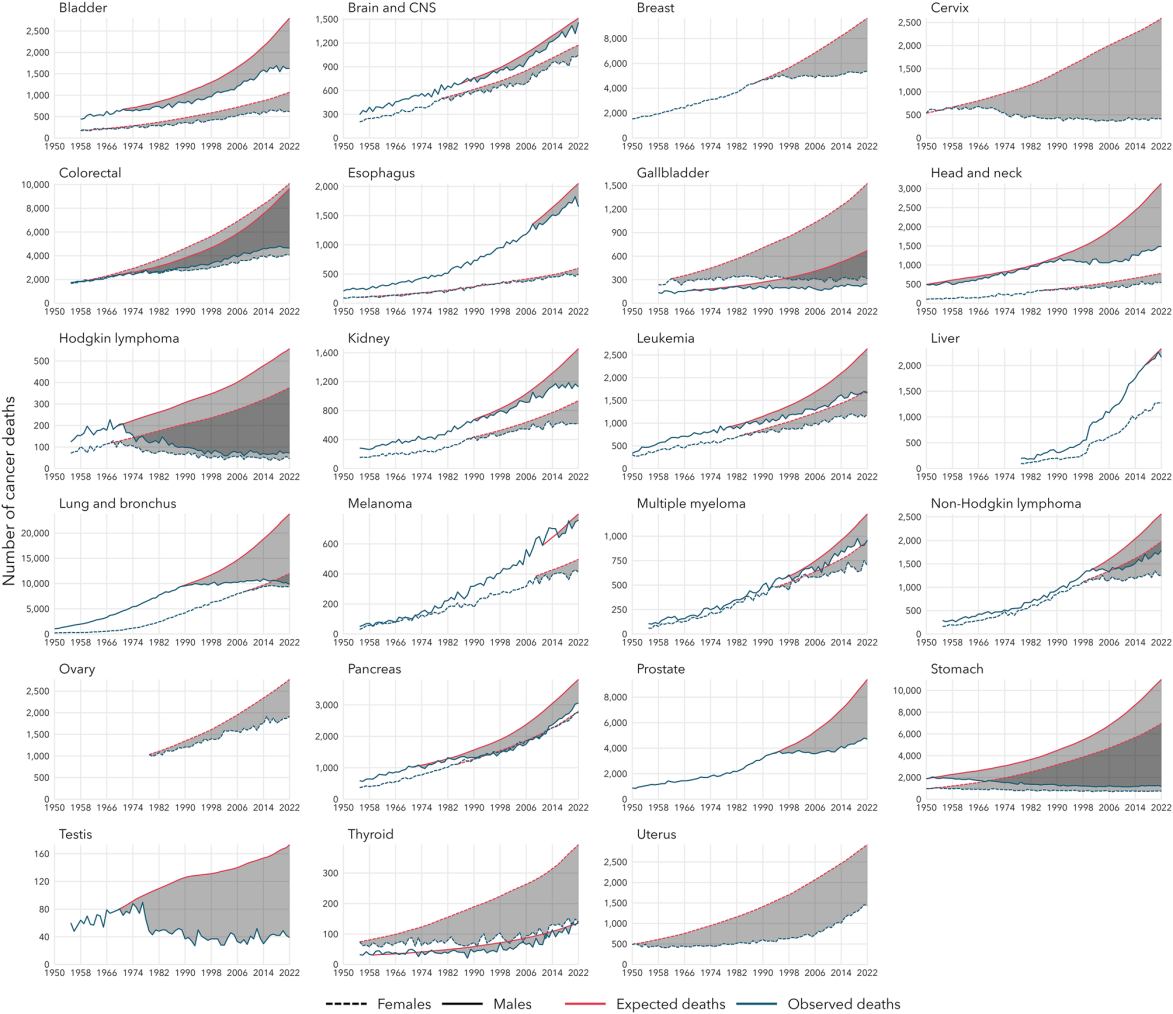
The observed (blue) and expected (red) number of cancer deaths for each cancer type in the period since cancer-specific age-standardized mortality rates peaked from 1950 to 2022, separately by sex. The shaded grey area represents the number of cancer deaths avoided since mortality rates peaked.

The number of observed, expected, and avoided cancer deaths in the period following peak mortality for site-specific cancer mortality are presented in Table 1 and Fig. 3. The largest number of avoided deaths for any single cancer site was for stomach cancer, which, in this study, had peak mortality rates in the early 1950’s, with 246,615 and 170,946 avoided deaths for males and females, respectively. There have been a considerable number of avoided deaths among tobacco-associated cancers (417,561 stomach; 218,244 colorectal; 186,553 lung; 66,281 cervix; 32,732 head and neck; 27,713 bladder; 22,464 leukemia; 20,428 pancreas; 8863 kidney; 3876 esophagus; 290 liver). For female-specific cancers (breast, cervix, ovary, uterus), there have been 201,979 avoided deaths. For male-specific cancers (prostate, testis), there have been 64,200 avoided deaths. There have been 256,347 avoided deaths for cervical, breast, and colorectal cancers in the period since screening programs were implemented for these cancers in 1960, 1988, and 2007, respectively.

### Relative improvement in cancer mortality

**Figure 4:**
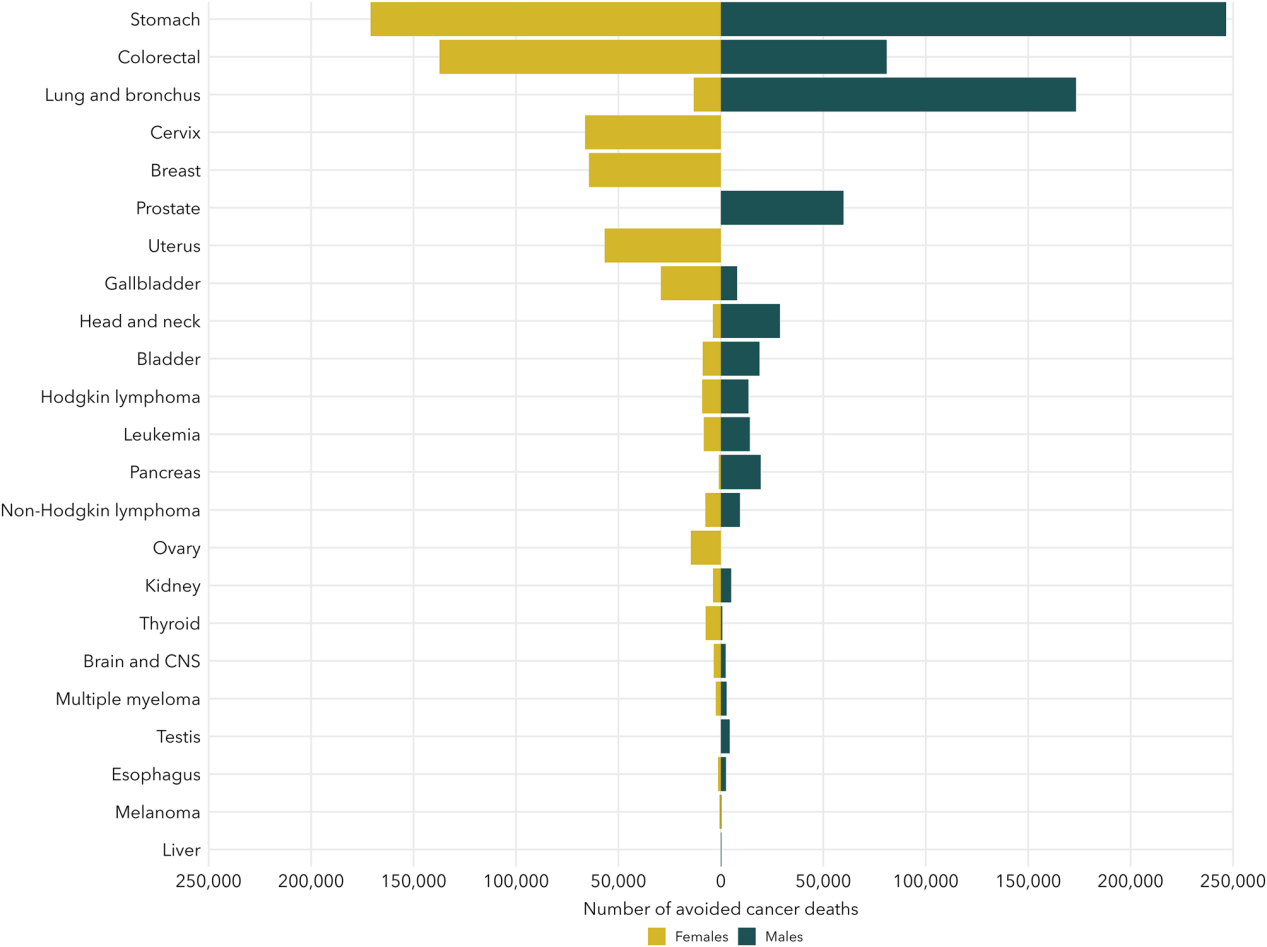
Number of avoided cancer deaths by sex and cancer site.

While the absolute number of deaths avoided in Canada is influenced by how common a cancer is, relative reductions in cancer mortality can highlight differences in progress by sex (see Fig. 4). There has been a 65% or larger reduction in stomach cancer and Hodgkin lymphoma mortality for both sexes and for cervical cancer among females. Since lung cancer mortality peaked in 1989 for males, there has been a 34% reduction in lung cancer mortality, however, for females, mortality rates peaked in 2008 and only an 9% reduction in mortality has been observed. Liver cancer mortality rates have yet to peak for females and we have not yet observed a significant reduction in melanoma mortality among males (SMR = 1.00, 95% CI 0.98–1.02).

Relative reductions in expected deaths are presented in Supplemental Fig. 3 and sex ratios for relative reductions are presented in Supplemental Fig. 4. In 2022, nearly 90% of the expected cancer deaths for cervix, Hodgkins lymphoma, and stomach cancer have been avoided. There are eight cancer sites that have yet to see more than a 25% reduction in expected deaths by 2022, this includes pancreas, multiple myeloma (males), lung and bronchus (females), esophagus, melanoma, thyroid (males), brain and CNS, and liver. While sex parity has been observed for the relative reduction in expected cancer deaths across several cancers, significant disparities still exist for cancers of the brain and CNS, esophagus, head and neck, liver, lung and bronchus, melanoma, pancreas, and thyroid.

## Discussion

We estimated the expected number of cancer deaths that would have occurred in a growing and aging population under a counterfactual scenario where mortality rates had remained at their peak. Major progress has been made both in terms of the absolute reduction in the number of deaths avoided, but also the relative proportion of mortality avoided. These analyses reflect that we are on our way to complete control for some cancers with nearly 90% reductions in mortality (Hodgkin lymphoma, stomach, cervix) and many more sites with more than 50% reductions. These analyses also highlight the cancer sites whereby more investment is needed to achieve progress. These findings give hope to future endeavors in cancer control to reduce cancer mortality where the direct impacts may be challenging to quantify within short time horizons.

Progress in reducing cancer deaths has been made through efforts across the continuum of cancer control. While a great deal of recent attention has been made to the clinical management of disease, from these analyses the greatest absolute impacts appear to have been made for sites through the primary and secondary prevention efforts. We highlight these impacts in the following sections.

### Primary prevention

Preventing cancers is undoubtedly the best way to reduce cancer mortality and has had the biggest influence on reductions in mortality over time. Changes in human behavior, such as the dramatic decreases in the use of salting and pickling in food preservation has led to a significant reduction of gastric/stomach cancers^10^. In the early twentieth century, a large majority of Canadians would regularly consume—often daily—foods that were pickled and salted/preserved with non-standardized methods. In recent decades, the identification of *Helicobacter pylori* (*H. pylori*) as a causal factor for stomach cancers and its treatment through antibiotics have also had a measurable effect^11^. Other dietary and lifestyle changes have likely played important roles in the incidence of many cancers. Unfortunately, melanoma has not had the same level of reduction in deaths as observed for many other cancers. The majority of melanomas are preventable by avoiding prolonged exposure to ultraviolet (UV) rays and this remains a priority for cancer control.

By far, the most successful cancer prevention strategy has been the anti-smoking/smoking cessation campaigns. Smoking has been associated with many malignancies, most notably cancers of the lung and bronchus^12^. We observed that more than 1 million cancers deaths were avoided among tobacco-associated malignancies. In Canada, smoking rates for men have been in decline since the 1960’s and for women since the 1980’s. In 1965, 61% of males smoked compared to 10% in 2020, while 37% of women smoked in 1979 compared to 12% in 2020^13^. This 20-year lag in smoking rates can be seen in the nearly 20-year lag in peak lung cancer mortality rates observed in this study. In Canada, we have only begun to see avoided lung cancer deaths in females compared to more than 170,000 avoided lung cancer deaths among males. This lag is partially attributable to the fact that smoking rates started at a much higher rate in men compared to women.

Nonetheless, there remains much to be accomplished in primary cancer prevention. Over the past two decades, the importance of body weight, physical activity, stress, diet, and alcohol consumption on cancer have emerged. These are challenging and multi-factorial exposures that are evolving over time with changing behavioral patterns and food supply systems. Continued research effort on these fronts are essential to improve our understanding on the complex interactions between host and environment to implement primary cancer prevention strategies effectively.

### Secondary prevention (screening and early detection)

Undoubtedly one of the great successes in cancer screening and early detection is the introduction of the Papanicolaou (Pap) test for cervical cancer screening of average-risk women. The Pap test was first discovered in 1928, and British Columbia was an early adopter by offering this service from 1948 onward^14^. British Columbia introduced the first provincial cervical cancer screening program in 1960^15^, with the test being partially subsidized in the early 1970’s then became freely available in 1984^16^. Cervical cancer screening has led to a drastic reduction in cervical cancer mortality among Canadian women and a substantial number of lives saved. In the coming decades, widespread HPV immunization will further reduce cervical cancer incidence and prevent subsequent cancer mortality with the ultimate goal of cervical cancer eradication as outlined by World Health Organization in their 2020 report^17^.

Breast cancer screening with mammography was the second population-focused screening test, first introduced in British Columbia in 1988, and by 2004, all provinces and territories (with the exception of Nunavut) had breast screening programs in Canada^18^. Canadian guidelines for colorectal cancer screening with fecal occult blood tests (FOBT) were first published in 2001 by the Canadian Task Force on Preventive Health Care, and in 2002 by the Public Health Agency of Canada^19^. Following the release of these guidelines, most provinces and territories mobilized to offer organized colorectal cancer screening, and the first provincial colorectal cancer screening programs became available in 2007, and in all 10 provinces by 2010^19^. In most jurisdictions, FOBT has since been replaced by the Fecal Immunochemical Test (FIT). Both breast and colorectal screening programs have led to improved early detection of cancers and reductions in cancer-specific mortality. Over the past few years, colorectal cancer has had the largest reductions in site-specific cancer incidence rates in Canada due to screening programs^2,20^. While screening uptake and adherence rates are not to the recommended levels, for all programs, the majority of eligible adults are being screened regularly and rates have remained relatively constant after a brief period of increasing uptake after program initiation^21^.

### Tertiary prevention (treatment and clinical management)

There have been substantial advances in treatment strategies for most cancers over the last several decades. Early improvements in cancer therapeutics were driven by the introduction of broad-spectrum systemic chemotherapies (e.g., platinum-based drugs like cisplatin, carboplatin, and oxaliplatin)^22^. In the years since then, drug-cancer specificity has increased significantly, leading to a proliferation of site-specific anti-cancer regimens. The use of hormone therapy (e.g., tamoxifen) to treat hormone-sensitive breast cancer, typically in combination with chemotherapy, is an example of a treatment breakthrough that has significantly reduced cancer mortality^23^. In addition, there has been a rapid growth in the number of molecularly targeted therapies that have become standard of care for many common cancers (e.g., EGFR TKIs for NSCLC)^24^. The approved use of Trastuzumab (Herceptin) for the treatment of HER2+ breast cancer in 1999 has similarly resulted in a large number of avoided breast cancer deaths.

More recently, the field of immuno-oncology (IO) with the advent of novel immunotherapies has led to another breakthrough in cancer treatment, the full benefit of which has not yet been realized. In the coming decades, the progress made in IO drug development may usher in the next chapter of cancer therapy. PD-1/PDL-1 inhibitors such as pembrolizumab, atezolizumab, and nivolumab, and to a lesser extent, other immune checkpoint inhibitors such as CTLA-4 (e.g., ipilimumab), have been success stories in improving lung cancer and melanoma survival and will continue to have an impact for years to come^25^. Overall, 5-year cancer survival rates have improved considerably since the 1990’s^26^, and it is anticipated that further developments in novel targeted and immunotherapies will have a further impact on outcomes across many cancers, including in the metastatic setting, where durable response or cures were not previously possible.

### Strengths and limitations

This study has a few limitations that are worth noting. First, the data for this study were derived from multiple sources and cancer definitions and coding have been updated over the study period. In general, the definitions were similar and the amount of bias is expected to be minimal. Second, the estimation for expected deaths implicitly assumes that the at-risk pool remains proportional over time. Lastly, many cancers have shared etiologies. Deaths from a given cancer necessarily preclude deaths from another cancer. Changes in risk factors over time may reduce deaths from multiple cancers and the number of avoided deaths based on aggregate-level data may be partially optimistic. To address this limitation, we have chosen not to sum avoided deaths across site-specific cancers. The mortality data were available back to 1950, however, for some cancers (e.g., stomach), cancer mortality rates have been declining since then and the actual peak mortality rates likely occurred earlier in the twentieth century (pre-1950). For these cancers, our estimates for the number of avoided deaths is likely an underestimate. Nonetheless, this is the largest and most comprehensive study, to date, in estimating the impact of cancer control on avoided cancer deaths in Canada. This study was based on over 70 years of detailed historical mortality data.

## Conclusions

Our study provides important insights into the progress made in reducing cancer mortality in Canada over the last several decades. The substantial reduction in the number of avoided cancer deaths highlights the importance of continued investment in cancer prevention, screening, and treatment efforts to further reduce the impact of cancer in Canada. While prevention is great fodder for discussion, historically, prevention-focused research has been comparatively under-funded. These analyses suggest that preventive measures for common exposures and effective low-cost screening tests have had the most dramatic impact on cancer mortality reductions, to date. Despite the increasing number of cancer deaths, for most cancers, mortality rates peaked many years ago and this has led to an avoidance of cancer deaths. This study highlights the great strides we have made in cancer control in Canada, and presents the substantial number of lives saved that can be attributed to the considerable advances made over the last several decades in prevention, screening, and treatment.

### Supplementary Information

Supplementary Information.

## Data Availability

The datasets analysed for the current study are available in the Global Cancer Observatory (https://gco.iarc.fr/overtime/en) and Statistics Canada (10.25318/1310014201-eng and 10.25318/1710000501-eng).
