# Supplementary material for: Progress in site-specific cancer mortality in Canada over the last 70 years
Source: Sci Rep. 2024 Mar 7;14:5688. doi: 10.1038/s41598-024-56150-x (PMC10920803; doi:10.1038/s41598-024-56150-x)
Supplement: Supplementary file 1 — Supplementary Information. [file 41598_2024_56150_MOESM1_ESM.pdf]

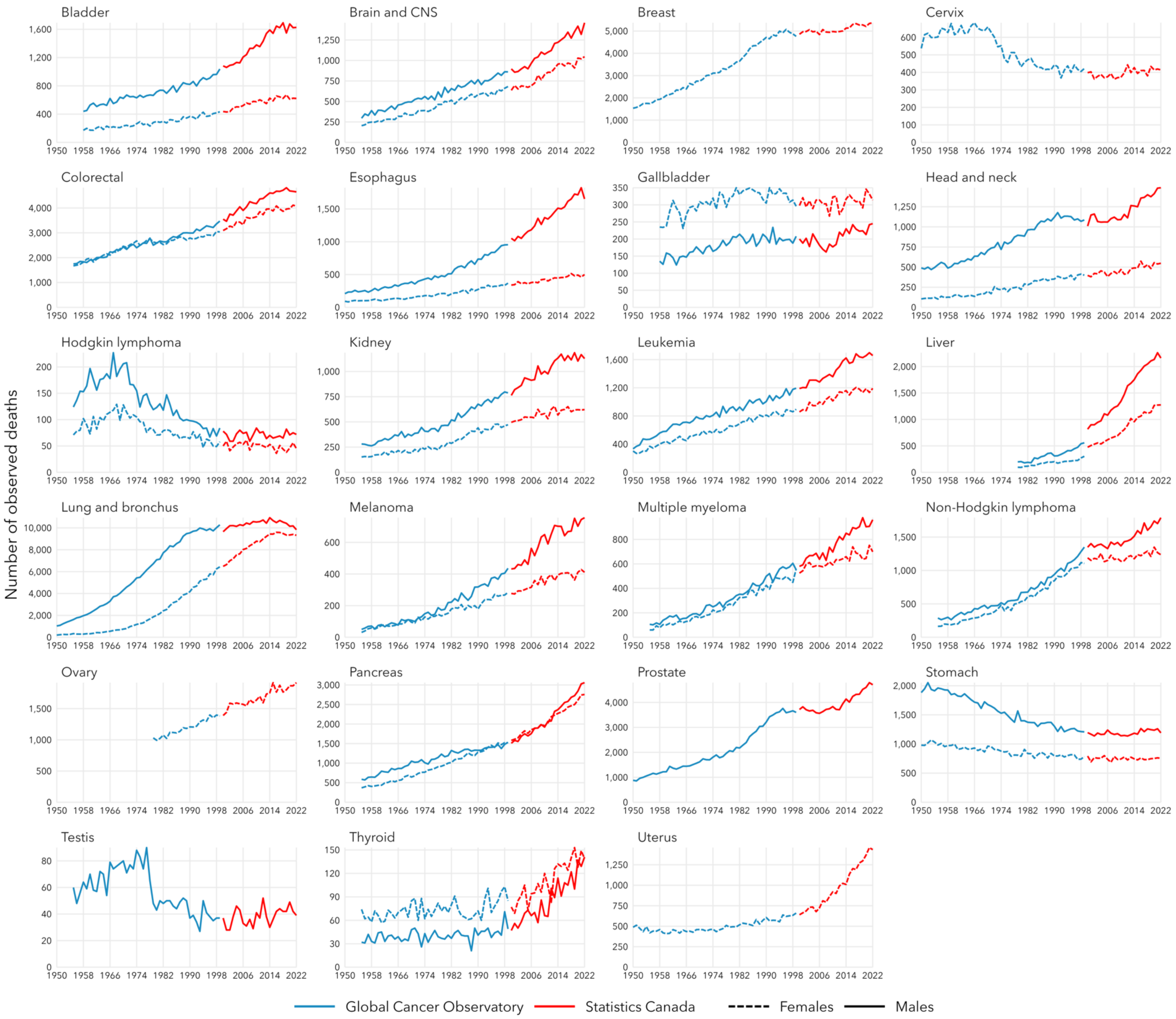

Supplemental Figure 1. Number of observed cancer deaths by site and sex based on the sources of cancer mortality data.

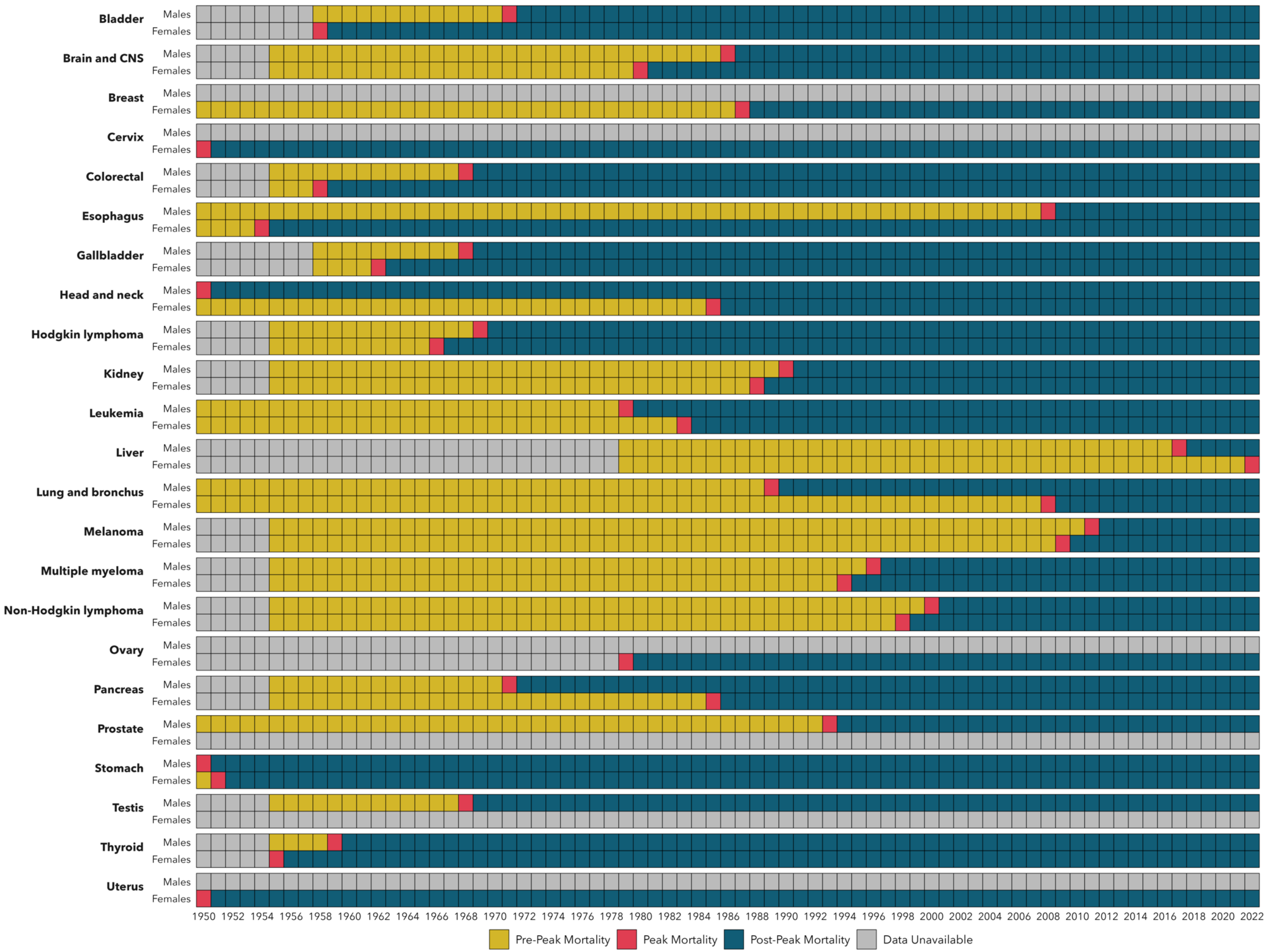

Supplemental Figure 2. Data availability based on sex and cancer site.

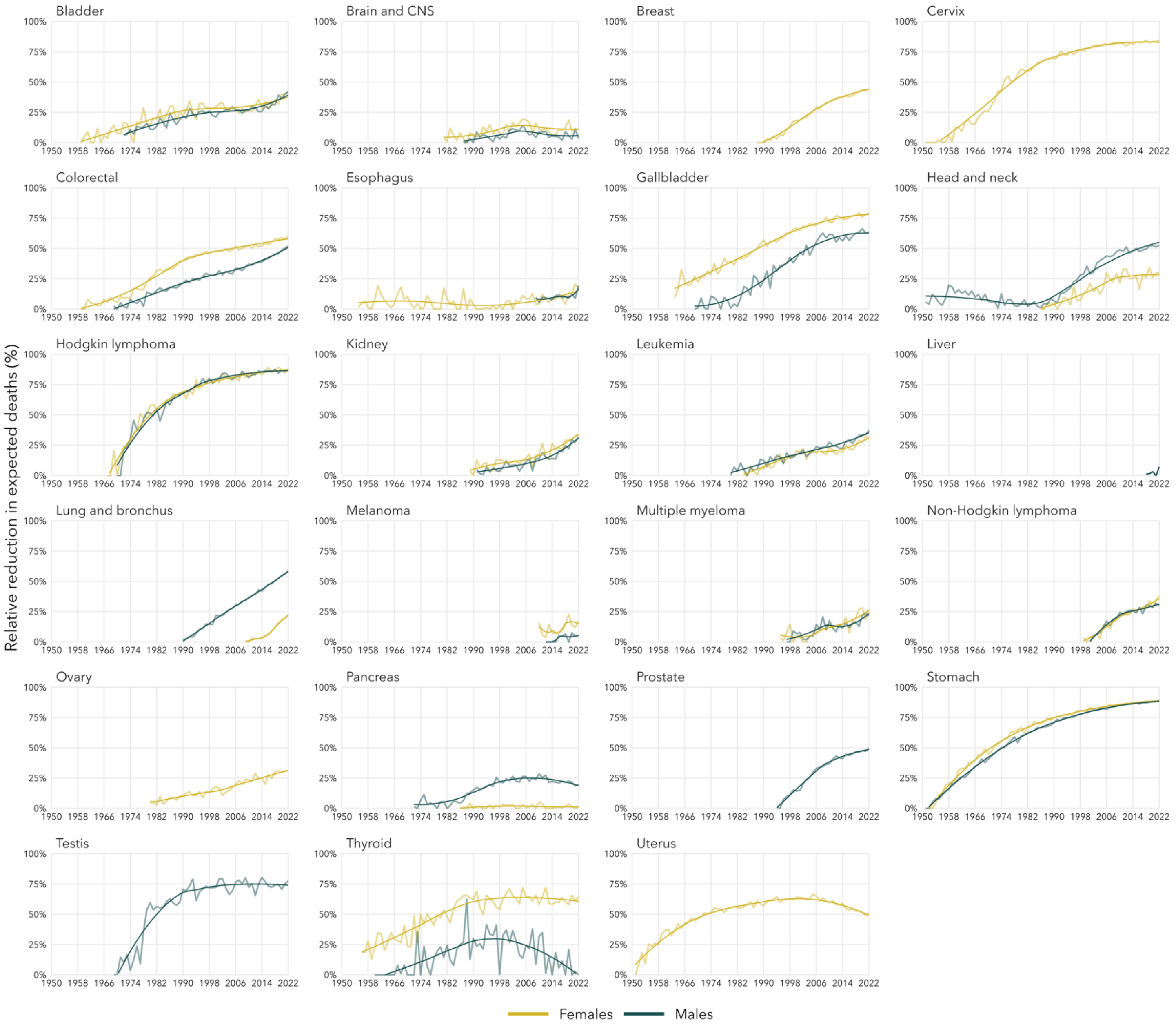

Supplemental Figure 3. Relative reduction in expected cancer deaths (avoided deaths / expected deaths) for males and females, by cancer site, in the period since site-specific age-standardized cancer mortality rates peaked. Local polynomial regressions (LOESS) were fitted to show smoothed relationships.

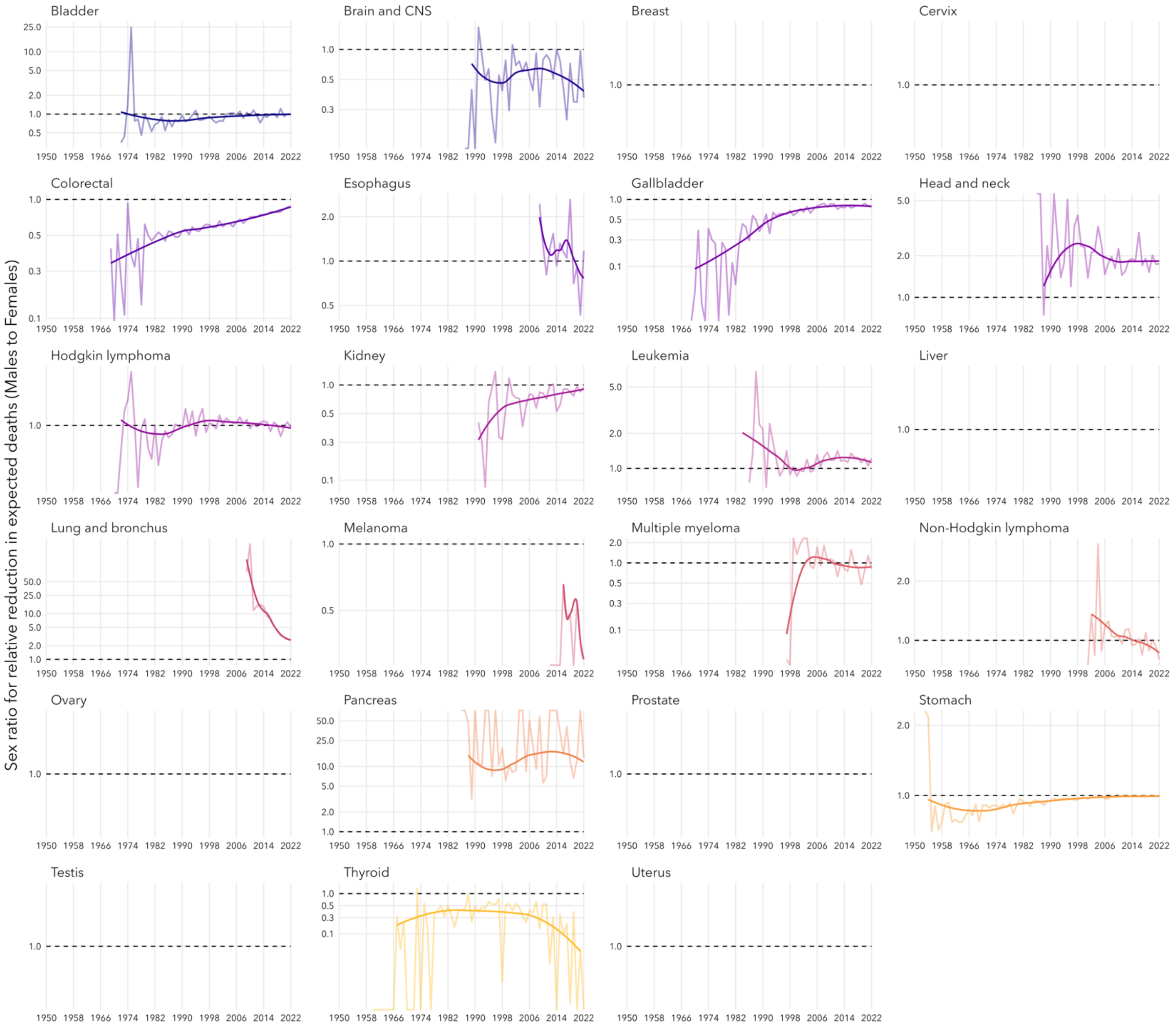

Supplemental Figure 4. Sex ratio (males to females) for the relative reduction in expected cancer deaths. A sex ratio above one indicates a larger reduction in expected deaths for males and a sex ratio below one indicates a larger reduction in expected deaths for females. A sex ratio of one indicates sex parity for the relative reduction in cancer deaths. Local polynomial regressions (LOESS) were fitted to show smoothed relationships.

Supplemental Table 1. International Classification of Disease (ICD, 10th revision) codes for each cancer site and data source.

Cancer	GLOBOCAN [1950-1999]	Statistics Canada [2000-2022]
All sites	C00-C96, C44	C00-C97
Bladder	C67	C67
Brain and CNS	C70-C72	C70-C72
Breast	C50	C50
Cervix	C53	C53
Colorectal	C18-C21	C18-C21
Esophagus	C15	C15
Gallbladder	C23-C24	C23-C24
Head and neck	C00-C14, C32	C00-C14, C32
Hodgkin lymphoma	C81	C81
Kidney	C64	C64-C65
Leukemia	C91-C95	C91-C95
Liver	C22	C22
Lung and bronchus	C33-C34	C33-C34
Melanoma	C43	C43
Multiple myeloma	C88, C90	C88, C90
Non-Hodgkin lymphoma	C82-C86, C96	C82-C86
Ovary	C56	C56
Pancreas	C25	C25
Prostate	C61	C61
Stomach	C16	C16
Testis	C62	C62
Thyroid	C73	C73
Uterus	C53-C55[a]	C54-C55

Abbreviations: CCS, Canadian Cancer Statistics; CNS, central nervous system.
[a] GLOBOCAN reported Uterus cancers including code C53 (Cervix) cancers. We subtracted C53 (Cervix) cancers from the Uterus cancer grouping to align with definitions for Statistics Canada.

Supplemental Table 2. 2011 Canadian standard population.

Age group	Population (No.)	Relative Weight (%)
**0-4**	1,899,064	5.53%
**5-9**	1,810,433	5.27%
**10-14**	1,918,164	5.59%
**15-19**	2,238,952	6.52%
**20-24**	2,354,354	6.86%
**25-29**	2,369,841	6.90%
**30-34**	2,327,955	6.78%
**35-39**	2,273,087	6.62%
**40-44**	2,385,918	6.95%
**45-49**	2,719,909	7.92%
**50-54**	2,691,260	7.84%
**55-59**	2,353,090	6.85%
**60-64**	2,050,443	5.97%
**65-69**	1,532,940	4.46%
**70-74**	1,153,822	3.36%
**75-79**	919,338	2.68%
**80-84**	701,140	2.04%
**85+**	643,070	1.87%
**Total**	**34,342,780**	**100.00%**

Note: The values for the 2011 are based on published data and not based on the population counts available through the Statistics Canada WDS API.
